# Association between sleep patterns and alcohol use disorders in workers

**DOI:** 10.1371/journal.pone.0308418

**Published:** 2024-08-06

**Authors:** Cho Rong Kim, Soo Young Kim, Jinhyun Kim, Eun-Cheol Park, Min Jin Ha

**Affiliations:** 1 Department of Health Policy Management, Graduate School of Public Health, Yonsei University, Seoul, Republic of Korea; 2 Institute of Health Services Research, Yonsei University, Seoul, Republic of Korea; 3 Department of Public Health, Graduate School, Yonsei University, Seoul, Republic of Korea; 4 Department of Psychiatry, Yonsei University College of Medicine, Seoul, Republic of Korea; 5 Department of Preventive Medicine, Yonsei University College of Medicine, Seoul, Republic of Korea; 6 Department of Health Informatics and Biostatistics, Graduate School of Public Health, Yonsei University, Seoul, Republic of Korea; University of Connecticut Health Center: UConn Health, UNITED STATES OF AMERICA

## Abstract

Alcohol use among workers that is intended to aid sleep may lead to alcohol use disorders. This study aimed to explore the association between sleep patterns and alcohol use disorders in workers. Data from the Korea National Health and Nutrition Examination Survey conducted in 2014, 2016, 2018, and 2020 were used for this study. We included only workers aged 19 years and older. The final analysis comprised 11,972 respondents (6,472 male and 5,500 female). Multiple logistic regression analysis was used to investigate the relationship between sleep patterns and alcohol use disorders. Workers with poor sleep patterns were more likely to develop alcohol use disorders compared to those with good sleep patterns (male: adjusted odds ratio [OR] 1.22, 95% confidence interval 1.07–1.39; female: adjusted OR 1.21, 95% CI 1.03–1.41). Workers with both poor sleep quality and less than seven hours of sleep had the highest odds of alcohol use disorders in both male (adjusted OR 1.73, 95% CI 1.38–2.17) and female (adjusted OR 1.44, 95% CI 1.13–1.84). Poor sleep patterns were associated with alcohol use disorders in male who work night shift (OR: 1.74, 95% CI: 1.25–2.42) and in female who worked more than 52 hours per week (adjusted OR: 1.71, 95% CI: 1.04–2.80). Customized sleep management programs should be provided to workers in sleep-deprived working environments to prevent them from developing alcohol use disorders.

## Introduction

Adequate sleep is essential for a healthy lifestyle for workers, but many workers struggle with insufficient sleep. In the United States, approximately 60% of workers experience sleep problems [1]. According to a recent study, workers struggle to get enough sleep for an average 5.3 days per month [2]. A study of South Korean workers found that short sleep duration of less than five hours was associated with high stress levels [3]. A cohort study in the United Kingdom found an increased risk of death among workers who slept for less than seven hours per night [4].

Sleep problems are considered a major risk factor for occupational injuries among workers [5], and the cost of lost productivity is in the tens of billions of dollars [6]. Fatigue due to sleep deprivation is one of the main causes of traffic accidents, especially among transportation workers [7]. Shift work increases the risk of fatigue-related errors and motor vehicle accidents while on the job or on the way home from work [8]. In addition, workers who work overtime are also at an increased risk of on-the-job injuries due to reduced sleep duration [9].

Work-related stress, caused by heavy workloads [10], long working hours [11], adverse working conditions [12], and workplace conflicts [13], detrimentally affects sleep quality and sleep deprivation. Sleep deprivation adversely impacts the physical and mental health of workers [14] and can reduce work efficiency [15]. Among workers, shift workers particularly face challenges with irregular schedules and circadian rhythm disruptions, increasing their susceptibility to sleep-related issues [16]. Good sleep habit is crucial for developing healthy lifestyle practices [17].

Individuals who are sleep deprived often rely on alcohol to aid sleep [18], which is a notable practice among shift workers [19, 20]. Studies on the general population have shown that the use of alcohol a sleep aid is common [21]. However, its use for sleep can gradually lead to harmful drinking habits [22]. Alcohol use disorders can adversely affect the health of workers [23] and negatively impact social relationships [24].

Previous studies have primarily focused on how excessive alcohol use causes sleep problems [25, 26]. However, it is worth noting that workers who experience sleep deprivation may rely on alcohol to induce sleep. Research focusing on the general working population in this regard is limited. Investigating the relationship between sleep and alcohol use disorders among workers is necessary for public health and can contribute to long-term health prevention. Therefore, this study aimed to investigate the association between sleep patterns and alcohol use disorders among workers, with the goal of developing strategies for effective sleep management in this population.

## Materials and methods

### Data and study population

We used data from the Korea National Health and Nutrition Examination Survey (KNHANES) conducted in 2014, 2016, 2018, and 2020. The KNHANES is a nationally approved statistical survey conducted annually by the Korea Centers for Disease Control and Prevention, and it aims to assess the health status and behaviors of the Korean population [27]. Regarding this survey, data were collected from residents of South Korea aged one year and above. Households are chosen as survey participants according to specific criteria for each survey area each year. The health interview and examination items used in this study were conducted and provided, respectively, by trained medical professionals and interviewers [27]. As the KNHANES adheres to the Declaration of Helsinki and offers de-identified data for public use, further ethical approval was not necessary for this study.

**Fig 1:**
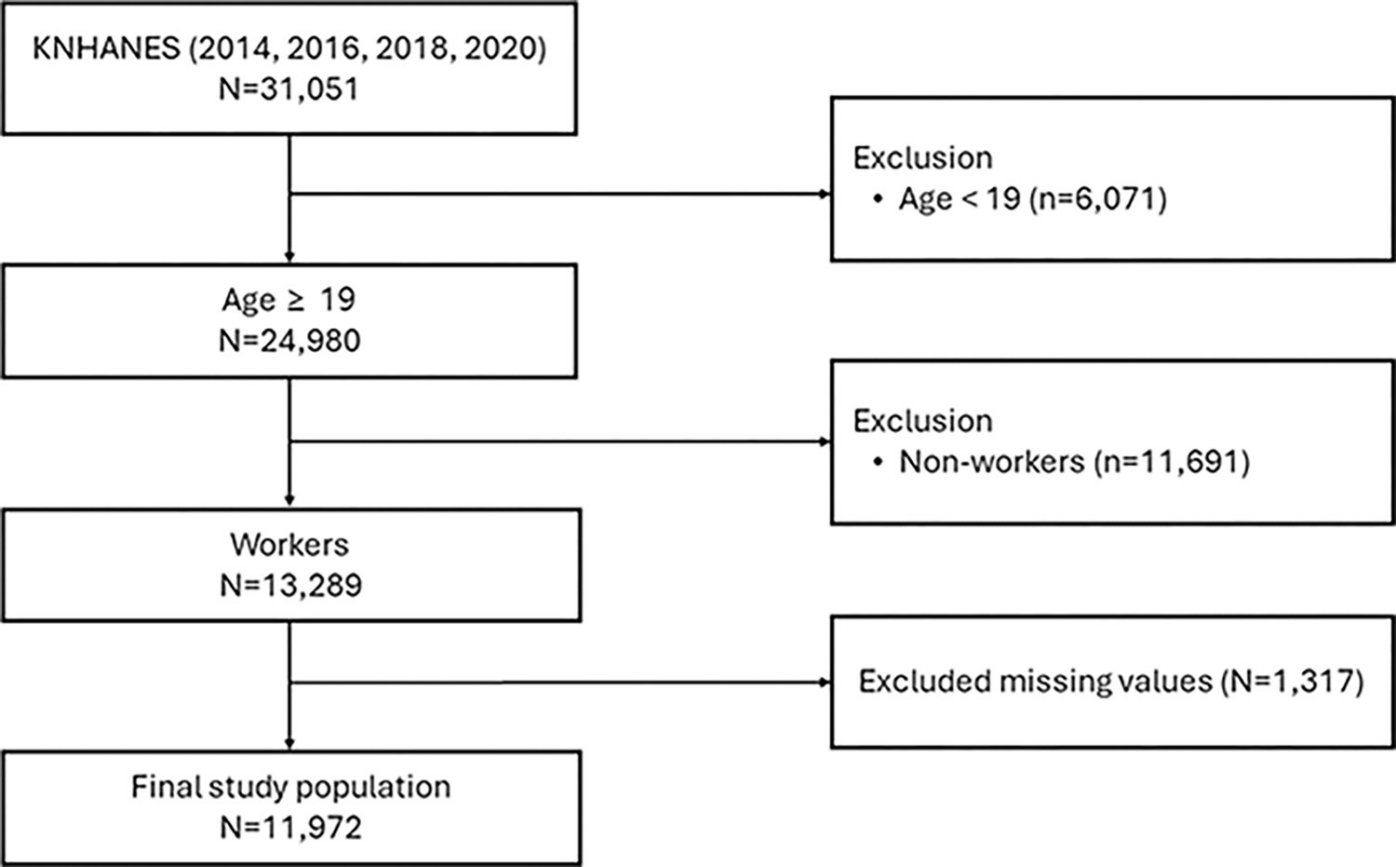
Flow chart of study participants.

The total survey population included 31,051 respondents over the four-year period of 2014, 2016, 2018, and 2020. The classification of workers included those who answered "yes" to the question in the survey, "In the past week, have you worked for more than one hour for income, or have you worked as an unpaid family worker for more than 18 hours?". We included the data on only workers aged 19 years and above in the analysis. Any missing values for each survey item were excluded from the analysis (N = 1,317). The final analysis included data on 11,972 (6,472 male and 5,500 female) individuals (Fig 1).

### Measures

The dependent variable in this study was alcohol use disorders as defined by the Alcohol use disorders identification test-consumption (AUDIT-C) questionnaire score. The AUDIT-C is an abbreviated version of the AUDIT, consists of the first three questionnaire of the AUDIT [28], and has high sensitivity and specificity in screening for alcohol use disorders [29]. The AUDIT-C includes the following three questions about alcohol consumption in the past year: (1) frequency of drinking, (2) amount of alcohol consumed on a single occasion, and (3) frequency of binge drinking, with each question scored on a 0–4-point scale, totaling a score of 12 [28]. As the standard alcohol drink differs in each country, the binge drinking questionnaire was based on the Korean standard, which defines binge drinking as more than seven drinks for male and more than five drinks for female in one drinking session, regardless of whether it being soju or hard liquor [30, 31]. In this study, we defined alcohol use disorders as an AUDIT-C score of 6 or more for male and 5 or more for female by referring to a previous study that measured thresholds using Korean data [32].

The independent variable was sleep patterns, which consisted of sleep quality and duration. Sleep quality was assessed using question 3 of the Patient Health Questionnaire-9, “In the past two weeks, have you had difficulty falling asleep, waking up for longer periods, or sleeping too much,” and only those who answered “not at all” were classified as having good sleep quality [33]. Sleep duration was defined as good if participants slept for seven or more hours, according to the recommended guidelines for optimal sleep duration in adults [34]. Data on sleep duration was collected using a survey questionnaire, from which sleep hours were calculated. A participant was classified as having good sleep patterns if both sleep quality and sleep duration were good, and if either was not satisfactory, the person was considered to have poor sleep patterns.

The covariates were demographic factors, included age (19–29, 30–39, 40–49, 50 or older), education level (elementary school or less, high school, college or higher), and marital status (married, single). Socioeconomic factors included and region of residence (metropolitan, middle and small cities) and household income level (low, mid, high). We also included occupational type (white collar, pink collar, blue collar), shift type (daytime, night shift) and working hours (less than 40 hours per week, 40–52 hours, and more than 52 hours per week) to reflect the diversity of work types based on previous research [35–37]. Health-related factors included smoking (yes, no), subjective health perception (low, mid, high), and perceived stress (low, high), and we further adjusted for whether respondents were advised by family members or doctors to cut back or stop drinking to control for the impact of alcohol use on sleep patterns.

### Statistical analysis

The chi-squared test was used to examine the general characteristics of the participants. Multiple logistic regression analysis was used to examine the association of sleep patterns with alcohol use disorders, controlling for covariates. Further subgroup analyses stratified by sex and key independent variables were performed. Multinomial logistic regression was used to analyze the association between workers’ sleep patterns and the severity of alcohol use disorders. The results of the analyses are expressed using odds ratios (ORs) and 95% confidence intervals (CIs). All statistical analyses were performed using SAS software version 9.4 (SAS Institute Inc., Cary, NC, USA), and a P-value<0.05 was considered statistically significant.

## Results

Table 1 presents the general characteristics of the respondents according to sex. The total sample was 11,972, of which 6,472 (54%) were male and 5,500 (46%) were female. Of the participants 55.6% (N = 3,599) of male and 51.8% (N = 3,353) of female had poor sleep patterns. Among workers with poor sleep patterns, alcohol use disorders were 57.7% (N = 2,076) for males and 29.5% (N = 988) for females. The association between sleep patterns and alcohol use disorders was significant in both males and females.

**Table 1:** General characteristics of the study population.

Variables	Male							Female						
	Alcohol use disorders (AUDIT-C^a^ ≥ 6)							Alcohol use disorders (AUDIT-C ≥ 5)						
	TOTAL		Yes		No		*P-value*	TOTAL		Yes		No		*P-value*
	N	%	N	%	N	%		N	%	N	%	N	%	
Total (N = 11,972)	6,472	100.0	3,559	55.0	2,913	45.0		5,500	100.0	1,530	27.8	3,970	72.2	
**Sleep patterns**							< .0001							0.001
Good	2,873	44.4	1,483	51.6	1,390	48.4		2,147	39.0	542	25.2	1,605	74.8	
Poor	3,599	55.6	2,076	57.7	1,523	42.3		3,353	61.0	988	29.5	2,365	70.5	
**Age**							< .0001							< .0001
19–29	654	10.1	390	59.6	264	40.4		843	15.3	434	51.5	409	48.5	
30–39	1,380	21.3	848	61.4	532	38.6		1,069	19.4	349	32.6	720	67.4	
40–49	1,522	23.5	925	60.8	597	39.2		1,343	24.4	379	28.2	964	71.8	
≥50	2,916	45.1	1,396	47.9	1,520	52.1		2,245	40.8	368	16.4	1,877	83.6	
**Education level**							< .0001							< .0001
Elementary school or less	464	7.2	206	44.4	258	55.6		664	12.1	103	15.5	561	84.5	
High school graduate or less	2,432	37.6	1,325	54.5	1,107	45.5		2,174	39.5	600	27.6	1,574	72.4	
College degree or higher	3,576	55.3	2,028	56.7	1,548	43.3		2,662	48.4	827	31.1	1,835	68.9	
**Marital status**							0.001							< .0001
Married	5,267	81.4	2,843	54.0	2,424	46.0		4,388	79.8	1021	23.3	3,367	76.7	
Single	1,205	18.6	716	59.4	489	40.6		1,112	20.2	509	45.8	603	54.2	
**Region**							0.841							0.758
Metropolitan cities	3,286	50.8	1,811	55.1	1,475	44.9		2,887	52.5	798	27.6	2,089	72.4	
Middle & small cities	3,186	49.2	1,748	54.9	1,438	45.1		2,613	47.5	732	28.0	1,881	72.0	
**Income level**							0.143							0.066
Low	983	15.2	520	52.9	463	47.1		893	16.2	268	30.0	625	70.0	
Mid	4,096	63.3	2,246	54.8	1,850	45.2		3,411	62.0	957	28.1	2,454	71.9	
High	1,393	21.5	793	56.9	600	43.1		1,196	21.7	305	25.5	891	74.5	
**Occupation**							< .0001							< .0001
White collar	2,627	40.6	1,487	56.6	1,140	43.4		2,405	43.7	714	29.7	1,691	70.3	
Pink collar	991	15.3	585	59.0	406	41.0		1,702	30.9	546	32.1	1,156	67.9	
Blue collar	2,854	44.1	1,487	52.1	1,367	47.9		1,393	25.3	270	19.4	1,123	80.6	
**Shift types**							0.880							0.000
Daytime workers	5,446	84.1	2,997	55.0	2,449	45.0		4,535	82.5	1213	26.7	3,322	73.3	
Night shift	1,026	15.9	562	54.8	464	45.2		965	17.5	317	32.8	648	67.2	
**Working hours**							0.001							< .0001
<40	1,708	26.4	882	51.6	826	48.4		2,581	46.9	627	24.3	1,954	75.7	
40–52	3,170	49.0	1,815	57.3	1,355	42.7		2,158	39.2	674	31.2	1,484	68.8	
>52	1,594	24.6	862	54.1	732	45.9		761	13.8	229	30.1	532	69.9	
**Smoking**							< .0001							< .0001
Yes	2,543	39.3	1,711	67.3	832	32.7		360	6.5	232	64.4	128	35.6	
No	3,929	60.7	1,848	47.0	2,081	53.0		5,140	93.5	1298	25.3	3,842	74.7	
**Subjective health perception**		0.0					0.004							< .0001
Low	841	13.0	435	51.7	406	48.3		859	15.6	199	23.2	660	76.8	
Mid	3,432	53.0	1,952	56.9	1,480	43.1		3,003	54.6	821	27.3	2,182	72.7	
High	2,199	34.0	1,172	53.3	1,027	46.7		1,638	29.8	510	31.1	1,128	68.9	
**Stress Perception**							< .0001							< .0001
Low	4,787	74.0	2,553	53.3	2,234	46.7		3,783	68.8	963	25.5	2,820	74.5	
High	1,685	26.0	1,006	59.7	679	40.3		1,717	31.2	567	33.0	1,150	67.0	
**Advised on Moderate Alcohol Consumption**							< .0001							< .0001
Yes	1,628	25.2	1,458	89.6	170	10.4		353	6.4	288	81.6	65	18.4	
No	4,844	74.8	2,101	43.4	2,743	56.6		5,147	93.6	1242	24.1	3,905	75.9	

^a^AUDIT-C: Alcohol Use Disorders Identification Test-Consumption.

Table 2 shows the results of the multiple regression analysis of the association between sleep patterns and alcohol use disorders after adjusting for covariates. Workers with poor sleep patterns had significantly higher odds of developing alcohol use disorder than those with good sleep patterns (male: adjusted OR 1.22 95% CI 1.07–1.39; female: adjusted OR 1.21, 95% CI 1.03–1.41).

**Table 2:** Results of factors associated with alcohol use disorders.

Variables	Male				Female			
	Alcohol use disorders (AUDIT-C^a^ ≥ 6)				Alcohol use disorders (AUDIT-C ≥ 5)			
	Adjusted OR^b^	95% CI^c^			Adjusted OR	95% CI		
**Sleep patterns**								
Good	1.00				1.00			
Poor	1.22	(1.07	-	1.39)	1.21	(1.03	-	1.41)
**Age**								
19–29	1.00				1.00			
30–39	0.97	(0.73	-	1.27)	0.57	(0.43	-	0.74)
40–49	0.78	(0.58	-	1.05)	0.41	(0.31	-	0.55)
≥50	0.55	(0.41	-	0.75)	0.21	(0.15	-	0.30)
**Education level**								
Elementary school or less	0.70	(0.50	-	0.97)	1.19	(0.81	-	1.75)
High school graduate or less	1.05	(0.89	-	1.23)	1.38	(1.10	-	1.72)
College degree or higher	1.00				1.00			
**Marital status**								
Married	1.00				1.00			
Single	0.99	(0.81	-	1.21)	1.15	(0.90	-	1.47)
**Region**								
Metropolitan cities	1.00				1.00			
Middle & small cities	0.98	(0.86	-	1.13)	0.99	(0.84	-	1.17)
**Income level**								
Low	0.73	(0.58	-	0.92)	1.20	(0.92	-	1.56)
Mid	0.89	(0.75	-	1.05)	1.21	(1.00	-	1.47)
High	1.00				1.00			
**Occupation**								
White collar	1.00				1.00			
Pink collar	1.11	(0.91	-	1.34)	1.29	(1.05	-	1.58)
Blue collar	0.97	(0.82	-	1.13)	1.04	(0.80	-	1.35)
**Shift types**								
Daytime workers	1.00				1.00			
Night shift	0.91	(0.76	-	1.09)	1.13	(0.93	-	1.38)
**Working hours**								
<40	1.00				1.00			
40–52	0.92	(0.78	-	1.08)	1.26	(1.06	-	1.51)
>52	0.82	(0.68	-	1.00)	1.42	(1.12	-	1.79)
**Smoking**								
Yes	2.10	(1.83	-	2.40)	3.38	(2.58	-	4.43)
No	1.00				1.00			
**Subjective health perception**								
Low	0.74	(0.59	-	0.92)	0.52	(0.40	-	0.68)
Mid	0.94	(0.81	-	1.08)	0.71	(0.60	-	0.84)
High	1.00				1.00			
**Stress Perception**								
Low	1.00				1.00			
High	0.92	(0.79	-	1.07)	1.08	(0.91	-	1.29)
**Advised on Moderate Alcohol Consumption**								
Yes	11.72	(9.58	-	14.35)	11.31	(7.92	-	16.15)
No	1.00				1.00			

^a^AUDIT-C: Alcohol Use Disorders Identification Test-Consumption.

^b^OR: odds ratio.

^c^CI: confidence interval.

Table 3 provides the results of the multiple regression analysis with subgroups stratified by the independent variables. Male shift workers with poor sleep patterns showed the highest association with alcohol use disorders (adjusted OR 1.74, 95% CI 1.25–2.42). Among female workers, poor sleep patterns were the most strongly associated with alcohol use disorders in those aged 50 years or older (OR 1.44, 95% CI 1.08–1.92), and in those working more than 52 hours per week (adjusted OR 1.71, 95% CI 1.04–2.80).

**Table 3:** The results of subgroup analysis stratified by independent variables.

Variables	Male					Female				
	Alcohol use disorders (AUDIT-C^a^ ≥ 6)					Alcohol use disorders (AUDIT-C ≥ 5)				
	Sleep patterns					Sleep patterns				
	Good	Poor				Good	Poor			
	Adjusted OR^b^	Adjusted OR	95% CI^c^			Adjusted OR	Adjusted OR	95% CI		
**Age**										
19–29	1.00	1.31	(0.89	-	1.94)	1.00	1.12	(0.78	-	1.60)
30–39	1.00	1.31	(0.89	-	1.94)	1.00	1.34	(0.96	-	1.86)
40–49	1.00	1.13	(0.89	-	1.43)	1.00	1.03	(0.76	-	1.38)
≥50	1.00	1.20	(0.98	-	1.46)	1.00	1.44	(1.08	-	1.92)
**Education level**										
Elementary school or less	1.00	1.67	(0.96	-	2.90)	1.00	1.32	(0.72	-	2.45)
High school graduate or less	1.00	1.30	(1.06	-	1.59)	1.00	1.16	(0.90	-	1.48)
College degree or higher	1.00	1.15	(0.98	-	1.37)	1.00	1.22	(0.99	-	1.50)
**Marital status**										
Married	1.00	1.23	(1.07	-	1.42)	1.00	1.28	(1.07	-	1.53)
Single	1.00	1.21	(0.90	-	1.64)	1.00	1.07	(0.78	-	1.48)
**Region**										
Metropolitan cities	1.00	1.27	(1.06	-	1.53)	1.00	1.22	(0.98	-	1.51)
Middle & small cities	1.00	1.19	(0.99	-	1.44)	1.00	1.19	(0.95	-	1.50)
**Income level**										
Low	1.00	1.13	(0.79	-	1.62)	1.00	1.12	(0.75	-	1.69)
Mid	1.00	1.23	(1.05	-	1.44)	1.00	1.29	(1.04	-	1.60)
High	1.00	1.23	(0.93	-	1.63)	1.00	1.02	(0.72	-	1.46)
**Occupation**										
White collar	1.00	1.18	(0.97	-	1.43)	1.00	1.15	(0.93	-	1.42)
Pink collar	1.00	1.38	(1.00	-	1.90)	1.00	1.28	(0.97	-	1.68)
Blue collar	1.00	1.22	(1.00	-	1.50)	1.00	1.30	(0.91	-	1.86)
**Shift types**										
Daytime workers	1.00	1.15	(1.00	-	1.33)	1.00	1.18	(0.99	-	1.40)
Night shift	1.00	1.74	(1.25	-	2.42)	1.00	1.32	(0.89	-	1.96)
**Working hours**										
<40	1.00	1.24	(0.95	-	1.61)	1.00	1.18	(0.93	-	1.49)
40–52	1.00	1.18	(1.00	-	1.40)	1.00	1.14	(0.90	-	1.43)
>52	1.00	1.29	(0.97	-	1.71)	1.00	1.71	(1.04	-	2.80)
**Smoking**										
Yes	1.00	1.22	(0.99	-	1.49)	1.00	1.97	(1.02	-	3.80)
No	1.00	1.24	(1.05	-	1.46)	1.00	1.18	(1.00	-	1.39)
**Subjective health perception**										
Low	1.00	1.22	(0.84	-	1.78)	1.00	1.15	(0.73	-	1.81)
Mid	1.00	1.34	(1.12	-	1.61)	1.00	1.31	(1.05	-	1.63)
High	1.00	1.07	(0.86	-	1.33)	1.00	1.08	(0.83	-	1.41)
**Stress Perception**										
Low	1.00	1.27	(1.10	-	1.48)	1.00	1.18	(0.97	-	1.43)
High	1.00	1.07	(0.82	-	1.41)	1.00	1.30	(0.97	-	1.75)
**Advised on Moderate Alcohol Consumption**										
Yes	1.00	1.87	(1.29	-	2.72)	1.00	1.63	(0.81	-	3.25)
No	1.00	1.17	(1.02	-	1.34)	1.00	1.19	(1.02	-	1.39)

^a^AUDIT-C: Alcohol Use Disorders Identification Test-Consumption.

^b^OR: odds ratio.

^c^CI: confidence interval.

**Fig 2:**
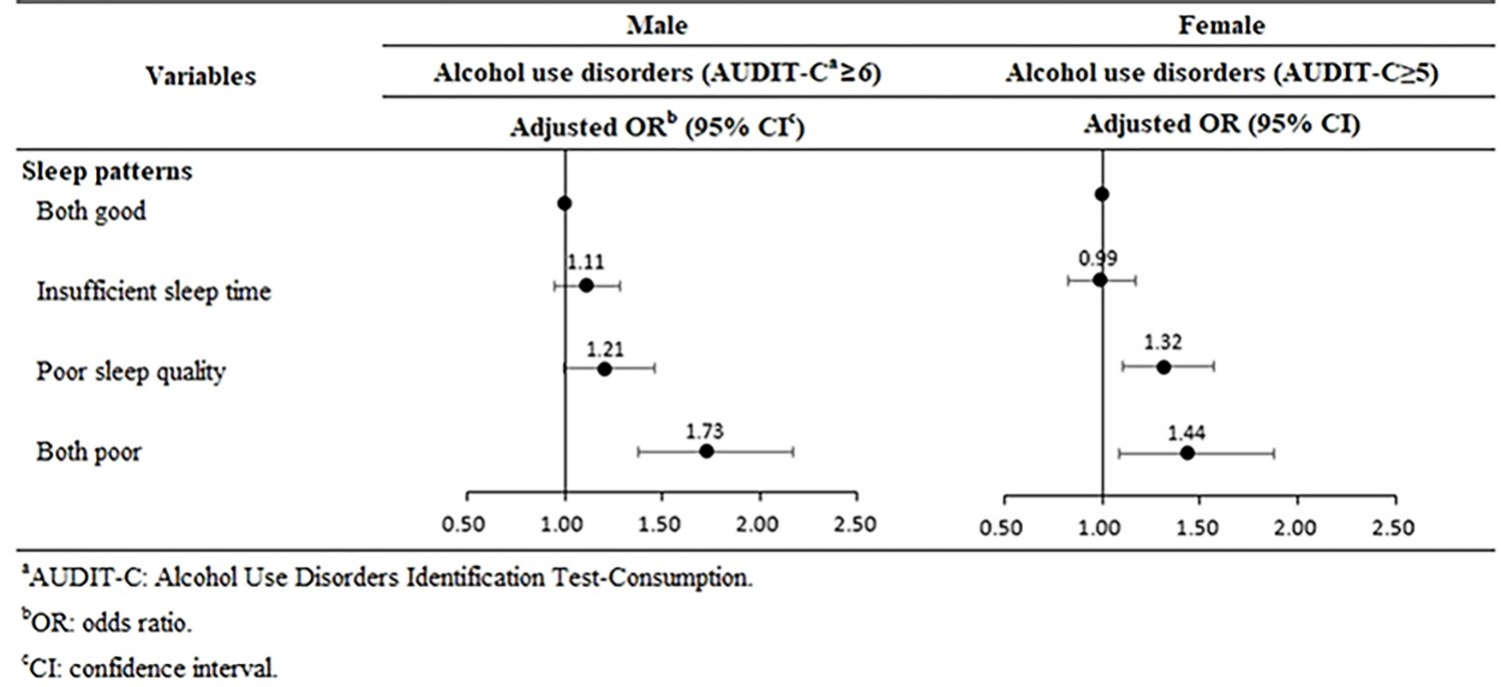
Results of subgroup analysis stratified by sleep duration and quality.

Fig 2 shows the results of the analysis of sleep patterns divided into sleep quality and sleep duration. Overall, workers with both poor sleep quality and poor sleep duration had increased odds of alcohol use disorders (male: adjusted OR 1.73, 95% CI 1.38–2.17; female: adjusted OR 1.44, 95% CI 1.13–1.84). Female workers had increased odds of alcohol use disorders even when only sleep quality was poor (adjusted OR 1.32, 95% CI 1.08–1.62).

Table 4 demonstrates the results of the multinomial logistic regression according to the severity of AUDIT-C score. Male workers with poor sleep patterns had higher odds of developing alcohol use disorders with increasing AUDIT-C scores than those with good sleep patterns (6–7 points: adjusted OR 1.10, 95% CI 0.93–1.30, 8–9 points: adjusted OR 1.26, 95% CI 1.07–1.49, and 10–12 points: adjusted OR 1.40, 95% CI 1.16–1.69). Female workers showed a tendency for increased odds of alcohol use disorders as their AUDIT-C scores increased, but this was only statistically significant at scores between 10 and 12 (5–7 points: adjusted OR 1.19, 95% CI 0.99–1.43, 8–9 points: adjusted OR 1.11, 95% CI 0.86–1.44, and 10–12 points: adjusted OR 1.73, 95% CI 1.17–2.54.

**Table 4:** The results of analysis stratified by severity of alcohol use disorders.

	Male													Female												
	Alcohol use disorders (AUDIT-C^a^ score)													Alcohol use disorders (AUDIT-C score)												
	No	6–7				8–9				10–12				No	5–7				8–9				10–12			
	Adjusted OR^b^	Adjusted OR	95% CI^c^			Adjusted OR	95% CI			Adjusted OR	95% CI			Adjusted OR	Adjusted OR	95% CI			Adjusted OR	95% CI			Adjusted OR	95% CI		
**Sleep patterns**																										
Good	1.00													1.00												
Poor		1.10	(0.93	-	1.30)	1.26	(1.07	-	1.49)	1.40	(1.16	-	1.69)		1.19	(0.99	-	1.43)	1.11	(0.86	-	1.44)	1.73	(1.17	-	2.54)

^a^AUDIT-C: Alcohol Use Disorders Identification Test-Consumption.

^b^OR: odds ratio.

^c^CI: confidence interval.

## Discussion

This study used nationally representative data to define sleep quality and sleep duration as components of sleep patterns and examined their association with alcohol use disorders. After accounting for adjustment variables, workers with poor sleep patterns showed a higher association with alcohol use disorders. These results were significant for both males and females and were particularly pronounced among workers with poor sleep quality and sleep duration of seven hours or less. Therefore, it is important to consider sleep health as a factor in reducing alcohol use disorders among workers and to actively consider ways to manage both sleep quality and duration.

Previous studies of workers have primarily focused on the impact of work stress on sleep quality and duration [38, 39]. Work stress has also been identified as a major factor leading to alcohol use [40]. However, this study sought to broaden the scope of worker healthcare by examining the link between poor sleep patterns and alcohol use disorders. In female workers in particular, a significant association with alcohol use disorders was observed even when sleep quality alone was poor. Females are more sensitive to sleep quality due to differences in role demands at home and work and vulnerability to stress than males, possibly increasing their susceptibility to alcohol use disorders [41, 42]. Additionally, female and older adults experience higher rates of severe insomnia [43]. In our study, poor sleep patterns were highly correlated with alcohol use disorders among female workers over the age of 50 years and older. Therefore, the need for understanding workers’ sleep habits in light of gender differences and intensive sleep hygiene education must be emphasized.

We investigated whether the association between sleep patterns and alcohol use disorders differs according to working conditions. In male workers with night shifts, the association between poor sleep patterns and alcohol use disorders was found to be the highest. Working night shifts can disrupt circadian rhythms and reduce sleep quality [44] and may be associated with the use of alcohol to cope. One study found that alcohol use among shift workers could exacerbate unstable circadian rhythms [22]. In addition, a previous study reported that working long hours was associated with alcohol use disorders [45]. However, this association was primarily significant among female workers in our study. Long working hours due to high job demands can limit workers’ sleep, which is important for recovery [46]. Given previous research showing increased alcohol consumption among female workers with high job demands [47], the stress of working long hours may be linked to alcohol use among female workers. Therefore, it is important to proactively implement sleep hygiene interventions for workers in sleep-deprived work environments to prevent the onset of alcohol use disorders.

It is important to identify high-risk workers early to prevent sleep problems from becoming chronic and leading to alcohol use disorders. Light alcohol consumption before bedtime can help induce sleep in the short term, but this effect diminishes over time and can have negative long-term effects [48]. High doses of alcohol can cause rapid eye movement sleep disruption, which can lead to a vicious cycle of sleep-related problems becoming chronic [49]. In addition, programs aiming to promote sleep hygiene in workers need interventions that take the work environment into consideration. Job stress and lack of support at work can contribute to sleep problems [50, 51], and active stress management programs are needed to manage these factors. This approach can help workers effectively manage workplace stress and maintain healthy sleep habits, contributing to their overall health.

This study has several limitations. First, we used cross-sectional data to identify associations between sleep patterns and alcohol use disorders. Therefore, it is difficult to infer temporal causality. Second, the data used in our study were self-reported, which may affect the accuracy of measures related to sleep duration and quality. Third, although we used reliable instruments to measure alcohol use disorders, their cut-offs varied across studies. Therefore, further research using data from multiple countries is needed to verify our study findings. Fourth, although we controlled for demographic, socioeconomic, and health-related factors in our study, there may still be unmeasured confounding variables that could affect the results. For example, psychiatric conditions such as anxiety and depression can have a significant impact on sleep and alcohol use. Individuals with these disorders may use alcohol as a coping mechanism, which can affect their sleep patterns. In addition, use of substance such as psychotropic medications can alter sleep architecture and affect alcohol metabolism. However, due to limitations of the secondary data, these variables were not considered.

## Conclusion

Poor sleep patterns among workers were associated with alcohol use disorders. Workers with poor sleep quality and less than seven hours of sleep were more likely to have alcohol use disorders. This association was higher among workers who worked night shifts or long hours, with differences according to gender. Therefore, customized sleep management programs tailored to workers with vulnerable working conditions are needed. Such programs should be designed considering gender-specific characteristics.

## Abbreviations

KNHANES: Korea National Health and Nutrition Examination Survey
AUDIT-C: Alcohol Use Disorders Identification Test-Consumption
OR: Odds Ratio
CI: Confidence Interval
